# The roles of tricellular tight junction protein lipolysis-stimulated lipoprotein receptor in malignancy of human endometrial cancer cells

**DOI:** 10.18632/oncotarget.8408

**Published:** 2016-03-28

**Authors:** Hiroshi Shimada, Seiro Satohisa, Takayuki Kohno, Syunta Takahashi, Tsubasa Hatakeyama, Takumi Konno, Mitsuhiro Tsujiwaki, Tsuyoshi Saito, Takashi Kojima

**Affiliations:** ^1^ Department of Obstetrics and Gynecology, Sapporo Medical University School of Medicine, Sapporo, Japan; ^2^ Department of Pathology, Sapporo Medical University School of Medicine, Sapporo, Japan; ^3^ Department of Cell Science, Research Institute for Frontier Medicine, Sapporo Medical University School of Medicine, Sapporo, Japan

**Keywords:** endometrial cancer, tricellular tight junctions, LSR, leptin, adiponectin

## Abstract

Lipolysis-stimulated lipoprotein receptor (LSR) has been identified as a novel molecular constituent of tricellular contacts that have a barrier function for the cellular sheet. LSR recruits tricellulin (TRIC), which is the first molecular component of tricellular tight junctions. Knockdown of LSR increases cell motility and invasion of certain cancer cells. However, the behavior and the roles of LSR in endometrial cancer remain unknown. In the present study, we investigated the behavior and roles of LSR in normal and endometrial cancer cells *in vivo* and *in vitro*. In endometriosis and endometrial cancer, LSR was observed not only in the subapical region but also throughout the lateral region as well as in normal endometrial epithelial cells in the secretory phase, and LSR in the cancer was reduced in correlation with the malignancy. Knockdown of LSR by the siRNA in cells of the endometrial cancer cell line Sawano, induced cell migration, invasion and proliferation, while TRIC relocalized from the tricellular region to the bicellular region at the membrane. In Sawano cells and normal HEEs, a decrease of LSR induced by leptin and an increase of LSR induced by adiponectin and the drugs for type 2 diabetes metformin and berberine were observed via distinct signaling pathways including JAK2/STAT. In Sawano cells, metformin and berberine prevented cell migration and invasion induced by downregulation of LSR by the siRNA and leptin treatment. The dissection of the mechanism in the downregulation of endometrial LSR during obesity is important in developing new diagnostic and therapy for endometrial cancer.

## INTRODUCTION

Endometrial cancer is the most common female genital malignancy in industrialized countries, and its incidence and mortality have recently been growing [1, 2]. In Japan, endometrial cancer incident and mortality in 2011 was about two times when compared with in 2001 [3]. Thus, new molecular targets for therapeutic approaches must be developed to improve the poor outcome by conventional treatment modalities.

The tight junction (TJ) is an epithelial cell-cell junction that regulates the flow of solutes through paracelluar pathways and maintains cell polarity [4, 5]. Tricellular tight junctions (tTJs) form at the convergence of bicellular tight junctions (bTJs) where three epithelial cells meet in polarized epithelia [6]. Lipolysis-stimulated lipoprotein receptor (LSR) was identified as a novel molecular constituent of tricellular contacts localized at most epithelial tissues [7]. LSR is required for formation of the normal tTJ, which has a strong barrier function for the cellular sheet. LSR recruits tricellulin (TRIC), which is the first molecular component of tTJs [6], and the interaction between the cytoplasmic domain of LSR and the C-terminal cytoplasmic domain of TRIC is required for this recruitment [7].

Loss of TJs compromises cellular polarity and stimulates dedifferentiation [8, 9]. Several studies have reported that loss of TJ proteins enhances tumor progression [10]. The tTJ protein TRIC is reduced in hepatic fibrolamellar carcinoma and tonsillar squamous cell carcinoma compared to normal tissues [11, 12]. Well-differentiated pancreatic ductal adenocarcinomas significantly overexpress TRIC as compared with poorly differentiated adenocarcinomas, and TRIC expression in the pancreatic cancer shows a significant negative correlation with the degree of differentiation [13]. Furthermore, TRIC expression in gastric carcinoma cells is negatively regulated by snail-induced epithelial-mesenchymal transition (EMT) [14]. Recently, the overexpression of some TJ proteins such as JAM-A and claudins have been shown to be associated with tumor growth and metastasis [15]. High TRIC expression in hepatocellular carcinomas and low TRIC expression in intrahepatic cholangiocarcinomas are correlated with poor prognosis [16]. Furthermore, high TRIC expression is associated with better survival in human hepatoblastoma [17]. It is thought that the tTJ protein LSR is also associated with tumor progression [18]. In fact, knockdown of LSR increases cell motility and invasion by bladder cancer cells [19]. However, the behavior and the roles of LSR in endometrial cancer remain unknown.

On the other hand, obesity, expressed as an increased body mass index (BMI), is associated with the risk of common cancers, including endometrial cancer [20–24]. In addition, adipokines, leptin and adiponectin, play important roles in the pathophysiology of cancer associated with obesity [25]. It is thought that circulating adiponectin, leptin and the adiponectin-leptin ratio may be risk factors for endometrial cancer [26]. Furthermore, metformin, which is one of the most common drugs for type 2 diabetes, reduces cancer-related mortality in patients with type 2 diabetes and the incidences of some cancers including endometrial cancer [27–29]. In addition, many studies indicate the anti-cancer effects of metformin *in vivo* and *in vitro* [30, 31]. The isoquinoline alkaloid berberine has an effects on type 2 diabetes like those of metformin [32], limiting the growth of various cancers [33–35]. However, the effects of adipokines, metformin and berberine on the function and expression of LSR in normal tissues and endometrial cancer remain unclear, though LSR was originally cloned as a candidate lipoprotein receptor [36].

In the present study, we investigated the behavior and roles of LSR in normal cells and endometrial cancer cells *in vivo* and *in vitro*. Furthermore, we focused on the endometrial malignancy related to obesity and investigated the effects of adipokines, metformin and berberine on the expression and the function of LSR in the cancer cells.

## RESULTS

### Distribution of lipolysis-stimulated receptor (LSR) and tricellulin (TRIC) in normal human endometrial tissues

**Figure 1:**
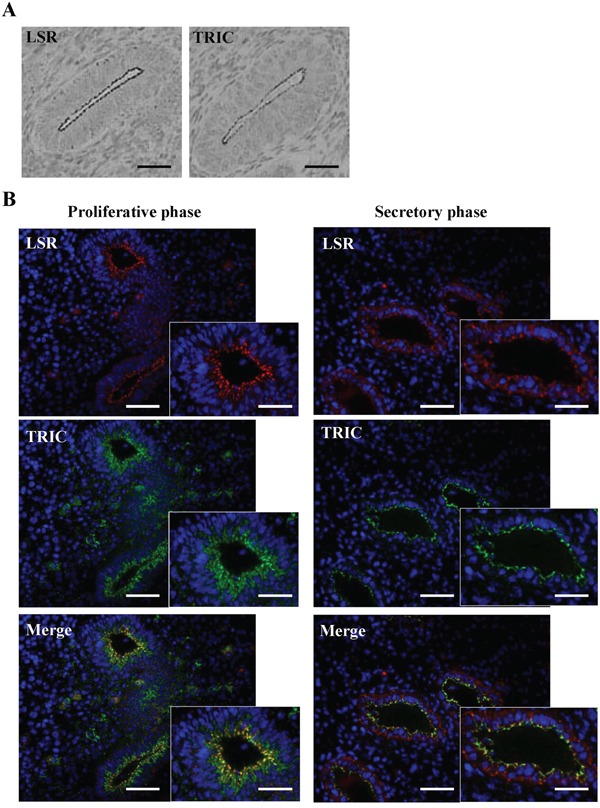
Expression of LSR and TRIC in human endometrial tissues **A.** Immunohistochemical staining for LSR and TRIC in human endometrial tissues. Scale bars: 100 μm. **B.** Fluorecent immunohistochemical staining for LSR (red) and TRIC (green) in human endometrium in the proliferative and secretory phases. Scale bars: 100 μm.

Immunohistochemical staining for LSR and TRIC was performed using the paraffin sections of normal human endometrial tissues. Both LSR and TRIC were localized in the subapical region of the endometrial epithelial cells (Figure 1A). Furthermore, since it is known that adherens and tight junction proteins in the endometrial epithelial cells are redistributed during the menstrual cycle [37, 38], the changes in the distribution of LSR and TRIC during the menstrual cycle were examined by fluorecent immunohistochemical staining using the cryosections. In the proliferative phase, LSR and TRIC were colocalized in the subapical region of the endometrial epithelial cells (Figure 1B). In the secretory phase, LSR was observed not only in the subapical region but throughout the lateral region, while TRIC was maintained in the subapical region (Figure 1B).

### Expression and localization of LSR and TRIC in endometriosis and endometrial carcinoma

**Figure 2:**
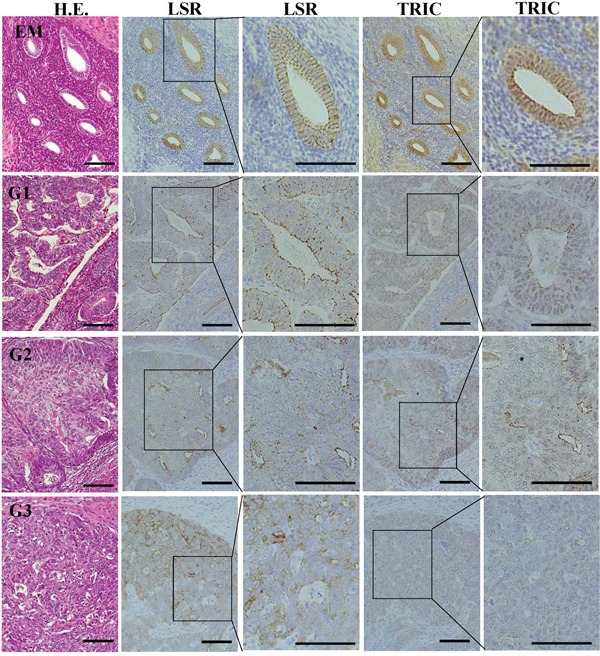
Expression and localization of LSR and TRIC in the tissues of endometriosis and endometrial cancer Hematoxylin and eosin (H.E.) staining and immunohistochemical staining for LSR and TRIC in the tissues of endometriosis (EM) and endometrial cancer (G1-G3). Third and fifth line figures are high magnifications of second and fourth line figures. Scale bar: 100 μm.

To investigate the distribution of LSR and TRIC during carcinogenesis of human endometrial cancer, immunohistchemical staining for LSR and TRIC was performed using the paraffin sections of endometriosis and endometrial cancer tissues. In endometriosis, LSR was observed not only in the subapical region but also throughout the lateral region, as with normal endometrial epithelial cells in the secretory phase, while TRIC was localized in the subapical region (Figure 2). In endometrial cancer which was diagnosis with the classic endometrial type I (endometrioid), LSR was highly expressed in some cancer cells that formed the gland-like structures and it was localized in both the subapical and lateral regions (Figure 2). Furthermore, LSR was reduced in G2 and G3 of the endometrial cancers compared to G1 (Figure 2). On the other hand, TRIC was reduced from G1 of the endometrial cancer (Figure 2).

### Expression and distribution of LSR and TRIC in human endometrial cancer cell lines

**Figure 3:**
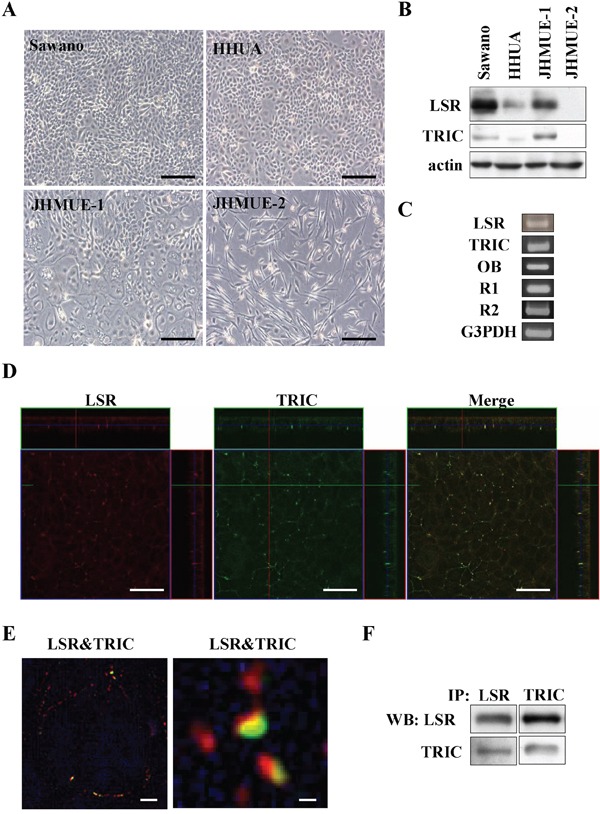
Expression and localization of LSR and TRIC in endometrial cancer cell lines **A.** Phase-contrast microscopy of endometrial cancer cells lines Sawano, HHUA, JHMUE-1, JHMUE-2. Scale bars: 100 μm. **B.** Western blotting for LSR and TRIC in Sawano, HHUA, JHMUE-1, and JHMUE-2 cells. **C.** RT-PCR for LSR, tricellulin, leptin receptor (OB) and adiponectin receptors (R1 and R2) in Sawano cells. **D.** Immunocytochemical staining for LSR (red) and TRIC (green) shown by a confocal laser microscopy in Sawano cells. Scale bars: 40 μm. **E.** Immunocytochemical staining for LSR (red) and TRIC (green) shown by super-resolution microscopy in Sawano cells. Scale bars: 1 μm. **F.** Immunoprecipitation dusing anti-LSR or anti-TRIC antibodies and Western blotting for LSR and TRIC in Sawano cells.

To study the roles and the regulation of LSR and TRIC in endometrial cancers in detail, we first investigated the expression and distribution of LSR and TRIC in endometrial cancer cell lines Sawano, HHUA, JHMUE-1 and JHMUE-2. In Sawano, HHUA and JHMUE-1, which exhibited epitheloid-like morphology, the proteins of LSR and TRIC were detected and LSR expression was higher level in Sawano cells than in the other cell lines in Western blotting (Figure 3A and 3B). In Sawano cells, not only the mRNAs of LSR and TRIC but also mRNAs of leptin receptor (OB) and adiponectin receptors (R1 and R2) were detected in RT-PCR (Figure 3C). LSR and TRIC were found to be colocalized at tricellular contacts in Sawano cells by confocal laser microscopy (Figure 3D). Super-resolution microscopy revealed that LSR and TRIC incompletely overlapped at tricellular contacts in Sawano cells (Figure 3E). In immunoprecipitation assays, immunoprecipitates using anti-LSR or anti-TRIC antibodies detected both LSR and TRIC (Figure 3F).

### Downregulation of LSR promotes cell migration, invasion and proliferation of Sawano cells

**Figure 4:**
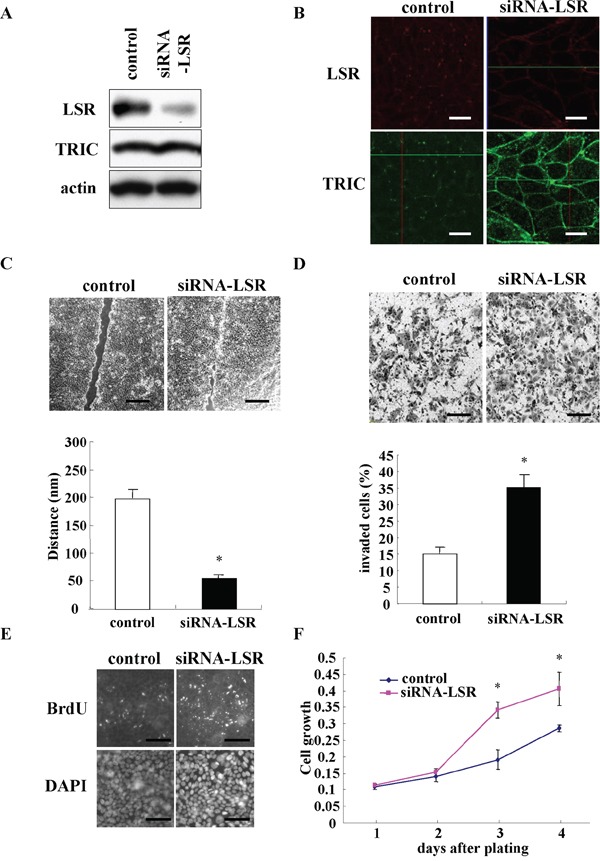
Knockdown of LSR by the siRNA promotes cell migration, invasion, and proliferation in Sawano cells **A.** Western blotting for LSR and TRIC in LSR-knockdown Sawano cells. **B.** Immunocytochemical staining for LSR (red) and TRIC (green) in LSR-knockdown Sawano cells. Scale bars: 20 μm. **C.** Migration assay in LSR knockdown Sawano cells. Scale bars: 400 μm. The results are shown as a bar graph. *p<0.01. **D.** Matrigel invasion assay in LSR-knockdown Sawano cells. Scale bars: 100 μm. The results are shown as a bar graph. *p<0.01. **E.** Immunocytochemical staining for bromodeoxyuridine (BrdU) and diamidino-2-phenylindole (DAPI) in LSR-knockdown Sawano cells. Scale bars: 100 μm. **F.** A line graph of cell proliferation assay in LSR-knockdown Sawano cells. *p<0.01.

To investigate the role of LSR in the endometrial cancer cells, knockdown of LSR was performed using the siRNA in Sawano cells. In Western blotting, LSR protein in Sawano cells was found to be decreased by knockdown of LSR, whereas no change of TRIC protein was observed (Figure 4A). In immunocytochemical staining after knockdown of LSR, LSR disappeared at the membranes and TRIC relocalized from the tricellular region to the bicellular region at the membrane (Figure 4B). Subsequently, to investigate whether LSR affected the endometrial cancer cell mobility, cell migration, invasion, and proliferation assays were performed using the LSR-knockdown Sawano cells. The downregulation of LSR markedly promoted cancer cell migration, invasion and proliferation compared to control cells (Figure 4C, 4D and 4E).

### Effects of leptin, adiponectin, metformin and berberine on LSR expression in Sawano cells

**Figure 5:**
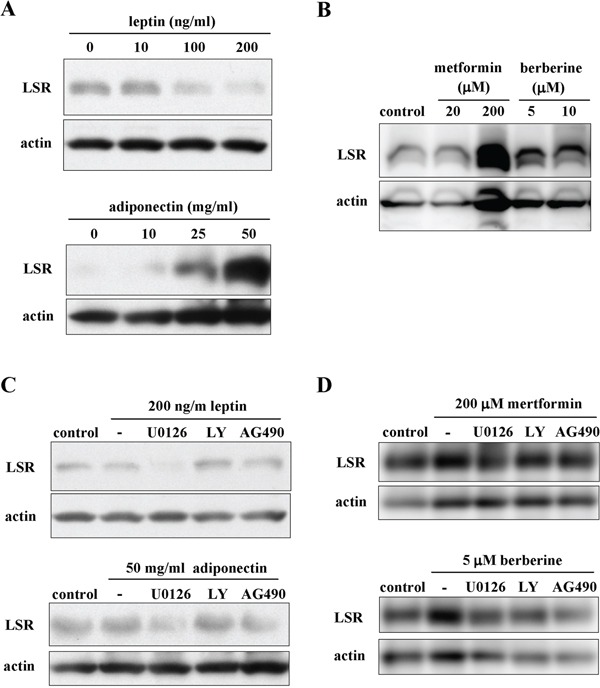
The changes of LSR expression indiced by leptin, adiponectin, metformin and berberine via a distinct signal transduction pathway in Sawano cells **A.** Western blotting for LSR in Sawano cells treated with leptin (0, 10, 100, 200 ng/ml) or adiponectin (0, 10, 25, 50 mg/ml) for 24 h. **B.** Western blotting for LSR in Sawano cells treated with metformin (20, 200 μM) or berberine (5, 10 μM) for 24h. **C.** Western blotting for LSR in Sawano cells pretreated with the inhibitor U0126, LY294002 or AG490, each at 10 μM, before treatment with leptin (200 ng/ml) or adiponectin (50 mg/ml) for 24 h. **D.** Western blotting for LSR in Sawano cells pretreated with the inhibitor U0126, LY294002 or AG490, each at 10 μM, before treatment with metformin (200 μM) or berberine (5 μM) for 24 h.

LSR is sensitive to high fat diets and its expression is regulated by circulating leptin [39]. However, little is known about the regulation of LSR expression in cancer cells. In the present study, we examined the effects of the lipid metabolism-associated substances leptin, adiponectin, metformin and berberine on LSR expression in Sawano cells. LSR expression of Sawano cells was reduced by treatment with leptin and increased by treatment with adiponectin in a dose-dependent manner (Figure 5A). Treatment with metformin and berberine induced LSR expression of Sawano cells in a dose-dependent manner (Figure 5B). Furthermore, to investigate which signal transduction pathways regulated the changes of LSR expression induced by leptin, adiponectin, metformin and berberine, Sawano cells were pretreated with the inhibitors of signaling pathways U0126 (MAPK inhibitor), LY294002 (PI3K inhibitor) and AG490 (JAK2/STAT inhibitor). Downregulation of LSR by leptin was inhibited by LY294002 and AG490 and upregulation of LSR by adiponectin was inhibited by U0126 and AG490 (Figure 5C). Upregulation of LSR by metformin was inhibited by U0126 and upregulation of LSR by berberine was inhibited by U0126, LY294002 and AG490 (Figure 5D).

### Metformin and berberine inhibit cell migration, invasion, and proliferation induced by LSR knockdown in Sawano cells

**Figure 6:**
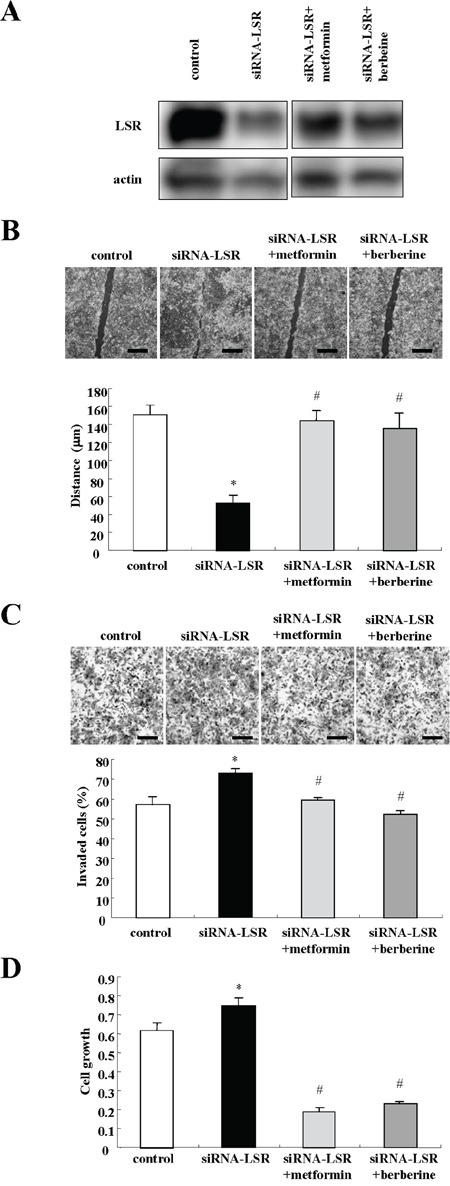
Metformin and berberine inhibit cell migration, invasion, and proliferation induced by LSR knockdown in Sawano cells **A.** Western blotting for LSR in LSR-knockdown Sawano cells treated with metformin (200 μM) or berberine (5 μM). **B.** Migration assay in LSR-knockdown Sawano cells treated with metformin (200 μM) or berberine (5 μM). Scale bars: 400 μm. The results are shown as a bar graph. Control vs. siRNA-LSR: *p<0.01. siRNA-LSR vs. siRNA-LSR+metformin or siRNA-LSR+berberine: #p<0.01. **C.** Matrigel invasion assay in LSR-knockdown Sawano cells treated with metformin (200 μM) or berberine (5 μM). Scale bars: 100 μm. The results are shown as a bar graph. Control vs siRNA-LSR: *p<0.01. siRNA-LSR vs. siRNA-LSR+metformin or siRNA-LSR+berberine: #p<0.01. **D.** A bar graph of cell proliferation assay in LSR-knockdown Sawano cells treated with metformin (200 μM) or berberine (5 μM). Control vs. siRNA-LSR: *p<0.01. siRNA-LSR vs siRNA-LSR+metformin or siRNA-LSR+berberine: #p<0.01.

To investigate whether metformin and berberine prevented the cell migration, invasion and proliferation induced by LSR knockdown, LSR Sawano with knockdown cells by the siRNA were treated with metformin and berberine. In Western blotting, the decrease of LSR expression caused by LSR knockdown was incompletely inhibited by metformin and berberine (Figure 6A). Treatment with metformin and berberine inhibited cell migration, invasion and proliferation induced by LSR knockdown, and they had a strong inhibitory effect on cell proliferation (Figure 6B, 6C and 6D).

### Metformin and berberine inhibit cell migration and invasion induced by leptin in Sawano cells

**Figure 7:**
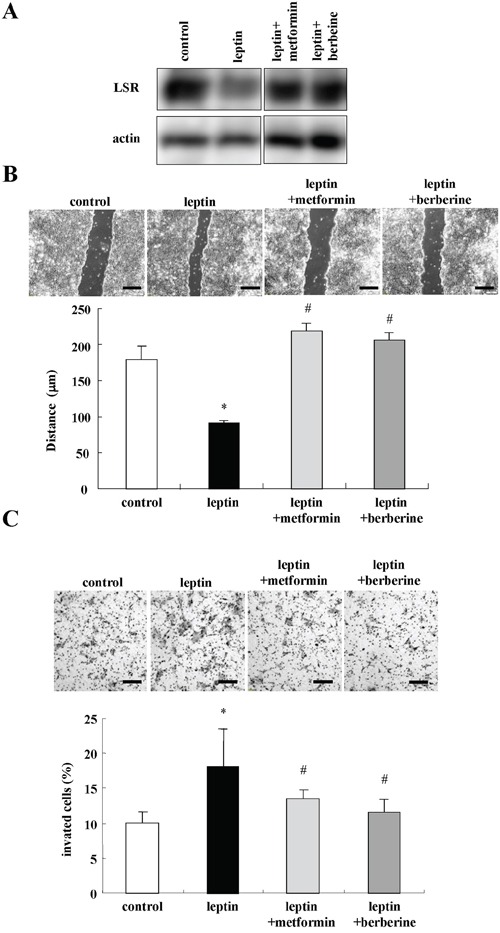
Metformin and berberine inhibit cell migration and invasion induced by leptin in Sawano cells **A.** Western blotting for LSR in Sawano cells pretreated with metformin (200 μM) or berberine (5 μM) before treatment with leptin (200 ng/ml) for 24h. **B.** Migration assay using Sawano cells pretreated with metformin (200 μM) or berberine (5 μM) before treatment with leptin (200 ng/ml) for 24h. Scale bars: 100 μm. The results are shown as a bar graph. Control vs. leptin: *p<0.01. Leptin vs leptin+metformin or leptin+berberine: #p<0.01. **C.** Matrigel invasion assay using Sawano cells pretreated with metformin (200 μM) or berberine (5 μM) before treatment with leptin (200 ng/ml) for 24h. Scale bars: 100 μm. The result is shown as a bar graph. Control vs. leptin: *p<0.05. Leptin vs. leptin+metformin or leptin+berberine: #p<0.01.

Leptin signaling promotes cell migration and invasive potential in various cancer cell lines [40–44]. To investigate whether metformin and berberine prevented the cell migration and invasion induced by leptin, Sawano cells were pretreated with metformin and berberine before treatment with leptin. In Western blotting, the decrease of LSR expression caused by leptin was completely inhibited by metformin and berberine (Figure 7A). Treatment with leptin promoted cell migration and invasion and metformin and berberine inhibited the cell migration and invasion induced by leptin (Figure 7B and 7C).

### Expression and distribution of LSR and TRIC in normal human endometrial epithelial (HEE) cells

**Figure 8:**
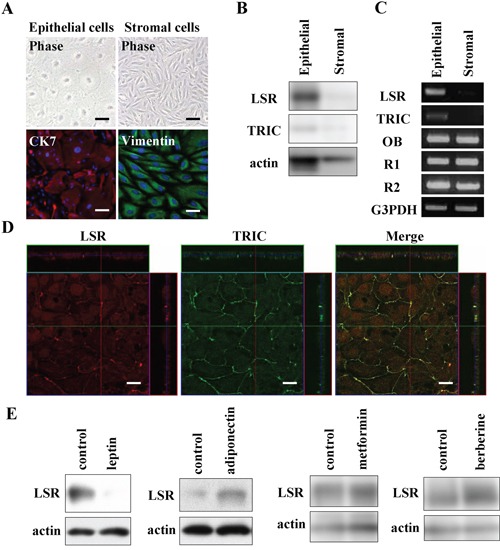
Expression and localization of LSR and TRIC in human endometrial epithelial (HEE) cells and the changes of LSR by leptin, adiponectin, metformin, and berberine **A.** Phase-contrast and immunocytochemical staining for cytokeratin 7 (CK7) and vimentin in HEE cells. Scale bars: 20 μm. **B.** Western blotting for LSR and TRIC in HEE cells and stromal cells. **C.** RT-PCR for LSR, TRIC, leptin receptor (OB) and adiponectin receptors (R1 and R2) in HEE cells and stromal cells. **D.** Immunocytochemical staining for LSR and tricellulin in HEE cells. Scale bar: 10 μm. **E.** Western blotting for LSR in HEE cells treated with leptin (200 ng/ml), adiponectin (50 mg/ml), metformin (200 μM), and berberine (5 μM).

To further investigate the regulation of LSR and TRIC in normal HEE cells we isolated and cultured epithelial cells and stromal cells from human endometrium. The epithelial cells showed a polygonal pattern and expressed CK7 as an epithelial marker but not vimentin as a mesenchymal cell marker, whereas the stromal cells were spindle-shaped like fibroblasts and expressed vimentin but not CK7 (Figure 8A). In Western blotting, proteins of LSR and TRIC were expressed in the epithelial cells, whereas in the stromal cells, none of these proteins were expressed (Figure 8B). In RT-PCR, the mRNAs of LSR and TRIC were detected only in HEE cells and the mRNAs of OB, R1 and R2 were detected in both HEE cells and stromal cells (Figure 8C). In immunostaining of the HEE cells, LSR and TRIC were found to be colocalized at tricellular contacts by a confocal laser microscopy (Figure 8D).

### The changes of LSR expression induced by leptin, adiponectin, metformin and berberine in normal HEE cells

We investigated whether the changes of LSR expression induced by leptin, adiponectin, metformin and berberine occurred in normal HEE cells. In Western blotting, downregulation of LSR by leptin and upregulation of LSR by adiponectin, metformin and berberine were observed as in Sawano cells (Figure 8E).

## DISCUSSION

In the present study, we determined the dynamic behavior and roles of LSR in normal and endometrial cancer cells *in vivo* and *in vitro*. In endometriosis and endometrial cancer, marked changes in the expression and distribution of LSR were observed correlating with the malignancy. In addition, the altered distribution of LSR in endometriosis and endometrial cancer was the same as that for normal HEEs in the secretory phase. LSR expression in endometrial cancer cells was reduced, correlating with a poor prognosis *in vivo* and *in vitro*. The altered expression of bTJ proteins such as occludin and claudins plays an important role in carcinogenesis [45]. However, little is known about the tTJ proteins TRIC and LSR. It is reported that ductal pancreatic adenocarcinoma and intrahepatic cholangiocarcinoma are associated with decreased TRIC expression, correlating with a poor prognosis [16]. Repression of TRIC expression is related to Snail-induced EMT in human gastric carcinoma [14]. TRIC expression in pancreatic cancer shows a significant negative correlation with the degree of differentiation [13]. Accordingly, it is thought that espression of tTJ protein LSR is also correlated with tumor progression and the degree of differentiation, like TRIC [18].

Furthermore, in the present study, downregulation of LSR by the siRNA and the leptin-treatment in endometrial cancer cells induced cell migration and invasion. Loss of the TJ proteins occludin and claudins enhances tumor progression via cell migration and invasion [10, 45]. It is reported that knockdown of LSR increases the cell motility and invasion of bladder cancer cells [18]. We recently found that, in cell of the well-differentiated pancreatic cancer cell line HPAC, knockdown of LSR by the siRNA also enhanced cell migration and invasion (personal data). It remains unclear how loss of transmembrane protein LSR enhances the cell migration, invasion and proliferation of endometrial cancer cells. It is thought that tumor suppressor angiomotin/merlin at TJ in epithelial cells, is directly linked to the pathogenesis of cancer via the hippo pathway [46]. In particular, angimotin/merlin removed from the TJ position induces TEAD/AREG via the YAP pathway and then enhances the cell migration, invasion and proliferation of cancer cells [47]. It is possible that the loss of LSR at tTJ may induce TEAD/AREG via the YAP pathway, because in DNA microarray and real-time PCR analyses of knockdown in Sawano cells by the siRNA, TEAD/AREG mRNAs were markedly increased compared to the control (data not shown). More recently, the development and progression of endometrial cancer via the hippo pathway was also reported [47]. In the near future, we will analyze the mechanisms of the YAP pathway.

Leptin promotes human endometriotic cell migration and invasion through the JAK2/STAT3 signaling pathway [48]. Leptin signaling via JAK2/STAT3 enhances cell invasion and promotes the metastasis of human pancreatic cancer [44]. Adiponectin inhibits leptin signalling via JAK2/STAT3 [49]. Adiponectin prevents the progression and development of some cancer cells [50, 51]. In the present study, in endometrial cancer cells and normal HEEs, a decrease of LSR induced by leptin and an increase of LSR induced by adiponectin were observed. In addition, in endometrial cancer cells, downregulation of LSR by leptin via PI3K and JAK2/STAT and upregulation of LSR by adiponectin via MAPK and JAK2/STAT were observed. Tight junction proteins are regulated by various cytokines and growth factors via distinct signal transduction pathways [52]. We have previously reported that JNK and NF-κB are largely involved in the regulation of tTJs, including TRIC expression and the barrier function [53]. LSR expression during endometrial carcinogenesis may in part be regulated by the adipokines leptin and adiponectin via distinct signaling pathways, including JAK2/STAT.

The adiponectin-leptin ratio is associated with the risk of obesity-related cancers such as, breast, colorectal, and pancreatic cancers [54–57]. Obesity is associated with earlier age at diagnosis of endometrioid-type endometrial cancers [58]. Furthermore, an increase in the adiponectin-leptin ratio reduces the risk of endometrial cancer [24, 59]. Liver-specific loss of LSR triggers systemic hyperlipidemia in mice and the downregulation of LSR is observed during obesity and/or weight gain [60]. As the relationship between LSR expression and obesity remains unclear in endometriosis and endometrial cancer, further study of the endometrial malignancy associated with obesity is needed.

The drugs for type 2 diabetes metformin and berberine enhanced LSR expression in endometrial cancer cells and normal HEEs and prevented the cell migration and invasion induced by downregulation of LSR in those treated with the siRNA or leptin in endometrial cancer cells. The AMPK activators metformin and berberine are thought to be new candidate therapeutic agents for endometrial cancer [59]. In the present study, in endometrial cancer cells, upregulation of LSR by metformin via MAPK and upregulation of LSR via MAPK, PI3K and JAK2/STAT were observed. Although there are thought to be anti-cancer effects of metformin and berberine via multiple mechanisms in endometrial cancer [61], the upregulation and maintenance of LSR expression by them is important in preventing obesity-related endometrial cancer.

Taken together, the results of this study provide novel evidence that tTJ protein LSR negatively regulates cancer cell progression and development in endometrial cancer. LSR expression in normal and endometrial cancer cells was regulated by the adipokines leptin and adiponectin. The mechanism in the regulation of endometrial LSR during obesity is important in developing new diagnostic and therapy for endometrial cancer. In addition, LSR is the host receptor for the binary toxin Clostridium difficile transferase (CDT), and the potential for using CDT as a therapeutic vector targeting LSR has been investigated in breast cancer [62, 63]. Thus, it is possible that LSR may be a potential targeting molecule in therapy for endometrial cancer like clostridium perfringens enterotoxin (CPE)-mediated therapy targeting claudin-4 [64, 65].

## MATERIALS AND METHODS

### Reagents and antibodies

A mouse monoclonal anti-cytokeratin 7 antibody, rabbit polyclonal anti-actin antibody and JAK2/STAT inhibitor (AG490) were obtained from Sigma-Aldrich (St. Louis, Mo., USA). Bromodeoxyuridine (BrdU) was obtained from Thermo Fisher Scientific (Waltham, MA). An anti-BrdU antibody was obtained from Dako (Tokyo, Japan). A rabbit polyclonal anti-vimentin (H-84) antibody was obtained from Santa Cruz Biotechnology (Santa Cruz, CA, USA). A PI3K inhibitor (LY294002) and mitogen-activated protein kinase (MAPK) inhibitor (U0126) were purchased from Calbiochem-Novabiochem Corporation (San Diego, CA). A mouse monoclonal anti-β-catenin antibody was obtained from BD Transduction Laboratories (San Jose, CA). Rabbit polyclonal anti-lipolysis-stimulated receptor (LSR) and anti-tricellulin (TRIC) antibodies were obtained from Zymed Laboratories (San Francisco, CA). Alexa 488 (green)-conjugated anti-rabbit IgG and Alexa 594 (red)-conjugated anti-mouse IgG antibodies were purchased from Molecular Probes, Inc. (Eugene, OR). Recombinant human leptin and recombinant human adiponectin were obtained from PeproTech (Rocky Hill, NJ). Metformin was purchased from Wako (Tokyo, Japan). Berberine was obtained from Tokyo Chemical Industry, Inc. (Tokyo, Japan).

### Cell line culture and treatment

The human endometrioid endometrial cancer cell line Sawano (RCB1152) was purchased from RIKEN Bio-Resource Center (Tsukuba, Japan). Sawano cells were maintained with MEM (Sigma-Aldrich) supplemented with 10% dialyzed fetal bovine serum (FBS; Invitrogen, Carlsbad, CA, USA). The medium contained 100 U/ml penicillin, 100 μg/ml streptomycin and 50 μg/ml amphotericin-B. Sawano cells were plated on 35- and 60-mm culture dishes, which were coated with rat tail collagen (500 μg dried tendon/ml in 0.1% acetic acid) and incubated in a humidified 5% CO_2_ incubator at 37°C. Sawano cells were treated with leptin (0, 10, 100, 200 ng/ml), adiponectin (0, 10, 25, 50 mg/ml), metformin (20, 200 μM), and berberine (5, 10 μM) with or without the inhibitors U0126, LY294002 or AG490, each at 10 μM.

### Isolation and culture of human endometrial epithelial (HEE) cells and stromal cells

Human endometrial tissues were obtained from patients with benign diseases (e.g., leiomyoma or adenomyosis uteri) who underwent hysterectomy in the Sapporo Medical University hospital. Informed consent was obtained from all patients, and the study was approved by the ethics committee of Sapporo Medical University. The human endometrial tissues were minced into pieces 2 to 3 mm^3^ in volume and washed with PBS containing 100 U/ml penicillin and 100 μg/ml streptomycin (Lonza Walkersville, Walkersville, MD) three times. These minced tissues were digested in 10 ml of Hanks' balanced salt solution with 0.5 μg/ml DNase I and 0.04 mg/ml Liberase (Roche, Basel, Switzerland) and then incubated with bubbling of mixed O_2_ gas containing 5.2% CO_2_ at 37°C for 20-30 min. The dissociated tissues were subsequently filtered with 300-μm mesh followed by filtration with 40-μm mesh (Cell Strainer, BD Biosciences, San Jose, CA). Stromal cells were removed by filtration, and the remaining cells were backwashed and collected as epithelial cells. After centrifugation at 1000*g* for 2 min, isolated cells were cultured in bronchial epithelial basal medium (BEBM, Lonza Walkersville) containing 4% fetal bovine serum (FBS) (CCB, Nichirei Bioscience, Tokyo, Japan) and supplemented with BEGM^®^ SingleQuots^®^ (Lonza Walkersville, including 0.4% bovine pituitary extract, 0.1% insulin, 0.1% hydrocortisone, 0.1% gentamicin, amphotericin-B [GA-1000], 0.1% retinoic acid, 0.1% transferrin, 0.1% triiodothyronine, 0.1% epinephrine, 0.1% human epidermal growth factor), 100 U/ml penicillin, 100 μg/ml streptomycin and 50 μg/ml amphotericin-B on 35- and 60-mm culture dishes or 24-well tissue culture plates (Corning Glass Works, Corning, N.Y., USA), coated with rat tail collagen (500 μg of dried tendon/ml of 0.1% acetic acid). Following the above protocol, tissue dissociation and cell isolation were repeated for the same sample three or four times. The cells were placed in a humidified 5% CO_2_:95% air incubator at 37°C. After 48 h, the bronchial epithelial basal medium containing 4% FBS was exchanged for medium without FBS. The epithelial cells were treated with leptin (200 ng/ml), adiponectin (50 mg/ml), metformin (200 μM) and berberine (5 μM).

### RNA interference and transfection

siRNA duplex oligonucleotides against LSR and tricellulin were synthesized by Thermo Fisher Scientific (Waltham, MA). At 24h after plating, Sawano cells were transfected with siRNAs of LSR and tricellulin using Lipofectamine™ RNAiMAX Reagent (Invitrogen).

### Immunohistochemical analysis

This study was approved by the ethics committee of the Sapporo Medical University School of Medicine. Human endometriosis and endometrial cancer tissues were embedded in paraffin after fixation with 10% formalin in PBS. Briefly, 5-μm-thick sections were dewaxed in xylene, rehydrated in ethanol, and heated with Vision BioSystems Bond Max using ER2 solution (Leica) in an autoclave for antigen retrieval. Endogenous peroxidase was blocked by incubation with 3% hydrogen peroxide in methanol for 10 min. The tissue sections were then washed twice with Tris-buffered saline (TBS) and preblocked with Block Ace for 1 h. After washing with TBS, the sections were incubated with anti-LSR (1:100) and anti-TRIC (1:100) antibodies for 1 h. The sections were then washed three times in TBS and incubated with Vision BioSystems Bond Polymer Refine Detection kit DS9800. After three washes in TBS, a diamino-benzidine tetrahydrochloride working solution was applied. Finally, the sections were counterstained with hematoxylin. Human endometrial carcinoma tissues and human endometriosis tissues were obtained from 6 patients with endometriosis and patients with 15 endometrial adenocarcinoma (G1: 7, G2: 4, or G3: 4) who underwent hysterectomy at Sapporo Medical University Hospital. The diagnoses of endometriosis and endometrial adenocarcinoma were established by both gynecologists and pathologists. All endometrial adenocarcinoma were the classic endometrial type I.

Human endometrial tissues from the proliferative and secretory phases were frozen in Neg-50 (Richard-Allan Scientific, Kalamazoo, MI, USA). Serial sections 7-8 μm thick were cut with a cryostat (Leica CM1850, Heidelberg, Germany) and placed on MAS-coated slides (Matsunami, Tokyo, Japan). The sections were incubated with rabbit polyclonal LSR and tricellulin antibodies (1:100) at room temperature for 1h. After washing with PBS, the sections were incubated with Alexa 488 (green)-conjugated anti-rabbit IgG or Alexa 584 (red)-conjugated anti-mouse IgG antibodies (1:200) at room temperature for 1 h. The specimens were examined using an epifluorescence microscope (Olympus, Tokyo, Japan). This study was approved by the ethic committees of the above institutions and the Sapporo Medical University School of Medicine.

### Immunocytochemical staining

The cultured cells in 35-mm glass-coated wells (Iwaki, Chiba, Japan) were fixed with cold acetone and ethanol (1:1) at −20°C for 10 min. After rinsing in PBS, the cells were incubated with anti-cytokeratin 7 (1:200), anti-LSR (1:100), anti-tricellulin (1:100), and anti-vimentin (1:100) antibodies at room temperature for 1 h. Alexa Fluor 488 (green)-conjugated anti-rabbit IgG and Alexa Fluor 594 (red)-conjugated anti-mouse IgG (Invitrogen) were used as secondary antibodies. The specimens were examined using an epifluorescence microscope (Olympus, Tokyo, Japan) and a confocal laser scanning microscope (LSM5; Carl Zeiss, Jena, Germany). Some images were captured using a Zeiss Elyra PS1 SIM equipped with a Zeiss Plan Apochromat inverted 63x/1.40 oil immersion objective lens using an Andor EM-CCD iXon 885 camera and a 1.6 x tube lens at room temperature (Carl Zeiss, Jena, Germany).

### RNA isolation and RT-PCR

Total RNA was extracted and purified using TRIzol (Invitrogen). Total RNA (1 μg) was reverse-transcribed into cDNA using a mixture of oligo (dT) and Superscript II reverse transcriptase according to the manufacturer's recommendations (Invitrogen). Synthesis of each cDNA was performed in a total volume of 20 μl for 50 min at 42°C and terminated by incubation for 15 min at 70°C. The polymerase chain reaction (PCR) was performed in a 20-μl mixture containing 100 pM primer pairs, 1.0 μl of the 20-μl total reverse transcription (RT) product, PCR buffer, dNTPs and Taq DNA polymerase according to the manufacturer's recommendations (Takara, Kyoto, Japan). Amplifications were carried out for 25–40 cycles depending on the PCR primer pair with cycle times of 15 s at 96°C, 30 s at 55°C and 60 s at 72°C. The final elongation time was 7 min at 72°C. Of the total 20-μl PCR product, 7 μl was analyzed by 1% agarose gel electrophoresis with ethidium bromide staining and standardized using a GeneRuler 100-bp DNA ladder (Fermentas, Ontario, Canada). The PCR primers used for LSR, tricellulin, leptin receptor (ObR), adiponectin receptor (R1 and R2), and glucose-3-phosphate dehydrogenase (G3PDH) by RT-PCR had the following sequences: LSR (sense 5′-CAGGACCTCAGAAGCCCCTGA-3′ and antisense 5′-AACAGCACTTGTCTGGGCAGC-3′), tricellulin (sense 5-TCAGACAGATGATGAGCGAGA-3′ and antisense 5-ATGTTCCTGTCGGCTTTCC-3′), leptin receptor (ObR) (sense 5′-AGGACGAAAGCCAGAGAC AACC-3′ and antisense 5′-GCCTGGGCCTCTATCTC CCA-3′), adiponectin receptor (R1) (sense 5′-TTCTTCC TCATGGCTGTGATGT-3′ and antisense 5′-AAGAAGC GCTCAGGAATTCG-3′), adiponectin receptor (R2) (sense 5′-ATAGGGCAGATAGGCTGGTTGA-3′ and antisense 5′-GGATCCGGGCAGCATACA-3′ and G3PDH (sense 5′-ACCACAGTCCATGCCATCAC-3′ and antisense 5′-TCCACCACCCTGTTGCTGTA-3′).

### Western blot analysis

The cultured cells were scraped from a 60 mm dish containing 400 or 600 ml of buffer (1mM NaHCO^3^ and 2mM phenylmethylsulfonyl fluoride), collected in microcentrifuge tubes, and then sonicated for 10s. The protein concentrations of the samples were determined using a BCA protein assay regent kit (Pierce Chemical Co.; Rockford, IL, USA). Aliquots of 15 μl of protein/lane for each sample were separated by electrophoresis in 5-20% SDS polyacrylamide gels (Wako, Osaka, Japan), and electrophoretic transfer to a nitrocellulose membrane (Immobilon; Millipore Co.; Bedford, UK) was performed. The membrane was saturated with blocking buffer (25mM Tris, pH 8.0, 125 mM NaCl, 0.1% Tween 20, and 4% skim milk) for over 30 min at room temperature and incubated with polyclonal rabbit anti-LSR (1:1000), anti-tricellulin (1:1000), and anti-actin (1:1000) antibodies at room temperature for over 1h. Then it was incubated with HRP-conjugated anti-mouse and anti-rabbit IgG antibodies at room temperature for 1h. The immunoreactive bands were detected using an ECL Western blot system.

### BrdU assay

To label cells in the S phase, BrdU (3 μg/ml) was added to the medium for 2 h. The cells were fixed with cold acetone and ethanol (1:1) at 20°C for 10 min. After being rinsed in PBS, the cells were preincubated with 2 N HCl at room temperature for 30 min before incubation with a mouse monoclonal anti-BrdU (1:100) antibody at room temperature for 1 h. The cells were incubated with Alexa Fluor 488 (green)-conjugated anti-mouse IgG (1:200) at room temperature for 1 h. Nuclei in the cells were counterstained with DAPI (Sigma-Aldrich). The BrdU-positive nuclei were counted using an epifluorescence microscope (Olympus).

### Proliferation assay

Sawano cells were seeded onto 96 well culture plates (Corning, NY, USA). Each day, the absorbance of three wells were measured using a Cell Counting Kit-8 (Wako, Osaka, Japan) according to manufacturer's instructions. The absorption at 450 nm was measured using an iMark Microplate Reader (Bio-Rad, Hercules, CA).

### Matrigel invasion assay

For the invasion assay, we used Matrigel (Becton Dickinson Labware, Bedford, MA) and Cell Culture Insert (pore size 8μm; Becton Dickinson Labware). Sawano cells were plated onto the upper chamber coated with Matrigel and the lower chamber of the Transwell was filled with human fibroblast conditioned medium containing 10 nM EGF, as an adhesive substrate. Then the cells were incubated for 36 h, after which the upper chamber was fixed with 100% methanol for 10 min and stained with Giemsa for 20 min. The areas of invading cells were measured using a microscope imaging system (Olympus, Tokyo, Japan).

### Migration assay

After the Sawano cells were plated onto the 35 mm dishes, they were cultured to confluence. At 24 h we wounded the cell layer using a plastic pipette tip (P200), and measured the length of the wound by using a microscope imaging system (Olympus, Tokyo, Japan).

### Immunopreciptitation

The dishes were washed with PBS twice and 300 μl of NP-40 lysis buffer (50 mM Tris–HCl, 2% NP-40, 0.25 mM Na-deoxycholate, 150 mM NaCl, 2 mM EGTA, 0.1 mM Na3VO4, 10 mM NaF, 2 mM PMSF) was added to the 60-mm dishes. The cells were scraped off, collected in microcentrifuge tubes and then sonicated for 10 s. Cell lysates were incubated with protein A-Sepharose CL-4B (Pharmacia LKB Biotechnology, Uppsala, Sweden) for 1 h at 4°C and then clarified by centrifugation at 15,000g for 10 min. The supernatants were incubated with the polyclonal anti-LSR antibody or anti-tricellulin antibody bound to protein A-Sepharose CL-4B overnight at 4°C. After incubation, immunoprecipitates were washed extensively with the same lysis buffer and subjected to Western blot analysis with anti-LSR and anti-tricellulin antibodies.

### Data analysis

Each set of results shown is representative of at least three separate experiments. Results are given as means ± SEM. Differences between groups were tested by analysis of variance followed by a post hoc test and an unpaired two-tailed Student's *t* test.
